# Hybrid acoustic metamaterial as super absorber for broadband low-frequency sound

**DOI:** 10.1038/srep43340

**Published:** 2017-02-27

**Authors:** Yufan Tang, Shuwei Ren, Han Meng, Fengxian Xin, Lixi Huang, Tianning Chen, Chuanzeng Zhang, Tian Jain Lu

**Affiliations:** 1 State Key Laboratory for Strength and Vibration of Mechanical Structures, Xi’an Jiaotong University, Xi’an 710049, P.R. China; 2 MOE Key Laboratory for Multifunctional Materials and Structures, Xi’an Jiaotong University, Xi’an 710049, P.R. China; 3 Department of Mechanical Engineering, The University of Hong Kong, Pokfulam Road, Hong Kong; 4 School of Mechanical Engineering, Xi’an Jiaotong University, Xi’an 710049, P.R. China; 5 Department of Civil Engineering, University of Siegen, Siegen 57068, Germany

## Abstract

A hybrid acoustic metamaterial is proposed as a new class of sound absorber, which exhibits superior broadband low-frequency sound absorption as well as excellent mechanical stiffness/strength. Based on the honeycomb-corrugation hybrid core (H-C hybrid core), we introduce perforations on both top facesheet and corrugation, forming perforated honeycomb-corrugation hybrid (PHCH) to gain super broadband low-frequency sound absorption. Applying the theory of micro-perforated panel (MPP), we establish a theoretical method to calculate the sound absorption coefficient of this new kind of metamaterial. Perfect sound absorption is found at just a few hundreds hertz with two-octave 0.5 absorption bandwidth. To verify this model, a finite element model is developed to calculate the absorption coefficient and analyze the viscous-thermal energy dissipation. It is found that viscous energy dissipation at perforation regions dominates the total energy consumed. This new kind of acoustic metamaterials show promising engineering applications, which can serve as multiple functional materials with extraordinary low-frequency sound absorption, excellent stiffness/strength and impact energy absorption.

Acoustic metamaterials are essentially artificial periodic structures that have extraordinary acoustic properties, such as broadband low-frequency absorption^1^,^2^,^3^,^4^, excellent sound insulation^5^,^6^ or enhanced sound transmission^7^,^8^. Moreover, acoustic metamaterials can be applied to other fields more than just sound absorption or transmission, such as high resolution edge detection^9^, far-field image magnification of subwavelength features^10^, imaging at a very deep subwavelength scale^11^, creating magnifying superlens^12^ and so on. This new kind of material exhibits unusual physical behaviors, of which negative effective density^13^,^14^,^15^, negative effective modulus^16^,^17^ and simultaneously negative density and modulus^18^,^19^,^20^,^21^ have been studied comprehensively. Zhu and coauthors innovate a unidirectionally transparent medium^22^, which can be applied to construct directional acoustic devices. More recently, they proposed a novel helical-structured acoustic metamaterial^23^, which can slow down acoustic waves at broad bandwidth. In the way to seek for lightweight structures having excellent low-frequency absorption performance^24^,^25^, it has long been a dilemma for gaining a broadband low frequency absorption and keeping a thin thickness of the structure. Metamaterials like porous lamella-crystals^3^ can achieve perfect absorption at around 500 Hz with a thickness of 0.5 m, and double layer platelet arrays with aluminum reflector^1^ can achieve perfect absorption at 164 Hz with 58 mm thickness. Recently, local resonant effects have been found and applied in ultrathin membrane metamaterials^26^,^27^, which have very low stiffness but can attain extraordinary absorption performance in broadband low-frequency range.

In this report, a new class of subwavelength acoustic metamaterial with broadband absorption in low frequencies is proposed by manipulating a lightweight sandwich plate with perforated honeycomb-corrugation hybrid core. As it has been demonstrated that such a hybrid-cored sandwich plate possesses excellent mechanical strength and impact energy absorption ability, whether the core is hexagonal^28^,^29^ or rectangular^30^,^31^, rectangular honeycombs are considered in the present study for simplicity. Small perforations introduced on both top facesheet and corrugated plate enable sound to penetrate into the structure while have negligible influence on its stiffness and strength. The series-parallel circuit analogue method is employed to establish an approximation theory to calculate the sound absorption coefficient of the novel sandwich structure, which is validated by comparing with finite element (FE) simulations. Perfect absorption has been found at 580 Hz, together with a two-octave 0.5 absorption bandwidth starting from 290 Hz, when the thickness of this metamaterial is just 60 mm. Comparison of sound absorption among perforated honeycomb-corrugation hybrid (PHCH), honeycomb corrugation hybrid (HCH) and honeycomb sandwich is performed, with the HCH and honeycomb sandwich only having perforation on top facesheet. Both viscous and thermal dissipations are investigated at the first resonant frequency, and it is found that viscous energy dissipation happening mainly at the perforation regions dominates the total energy consumed. These results indicate the significance of perforations on corrugation to enhance the sound absorption performance at low frequencies. Key parameters of different scales are analyzed to quantify the influence of plate thickness and perforation size on sound absorption.

## Theoretical Framework

**Figure 1:**
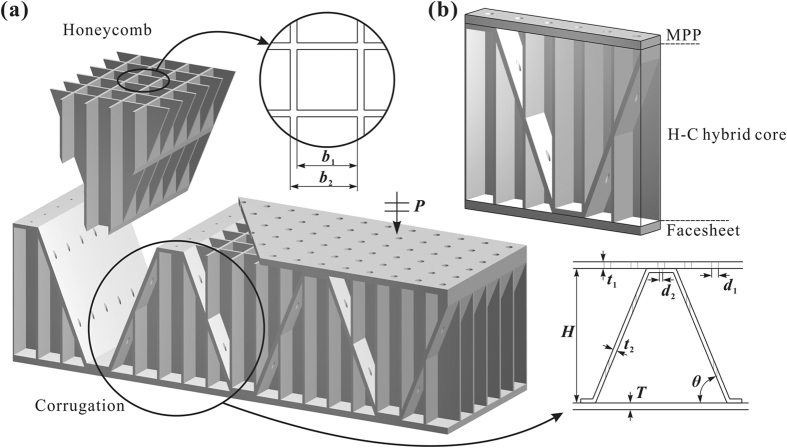
(**a**) Schematic of perforated honeycomb-corrugation hybrid (PHCH) metamaterial, which is composed of a micro-perforated panel as top facesheet, a honeycomb-corrugation hybrid as core and a panel as bottom facesheet. The corrugation is also perforated, just beneath the hole in top facesheet, but can have different perforation sizes. (**b**) One periodic unit cell. The front and right honeycomb walls are cut off to see details inside. Sound absorption of the metamaterial is investigated for a plane acoustic wave normally incident on top facesheet.

The proposed PHCH consists of two facesheets and a honeycomb-corrugation (H-C) hybrid core, as shown in Fig. 1. The top facesheet contains periodically distributed perforated holes as a micro-perforated panel (MPP), the honeycomb-corrugation hybrid core with perforated holes in the corrugation serves as a serial MPP panel, and the bottom facesheet as a rigid backing. This hybrid metamaterial has excellent mechanical stiffness and strength^28^,^29^, and hence is considered to be acoustically rigid in the following theoretical model. The proposed hybrid metamaterial is periodic, and the geometry of its unit cell is as follows. As shown in Fig. 1, the honeycomb core has a square cross-section with an inner side length *b*_1_ and a unit cell side length *b*_2_. The corrugation with an inclination angle *θ* has perforations in vertical direction. The thicknesses and perforation diameters of top facesheet and corrugation are *t*_1_, *t*_2_, *d*_1_ and *d*_2_, respectively. The thickness of H-C hybrid core is *H* and that of the bottom facesheet is *T*. The design of this novel class of metamaterial brings in series of different acoustic Helmholtz resonators comprised of narrow perforations and the cavity behind. When it reaches a resonant frequency, acoustic pressure variation in the cavity causes the plug of air in the perforation to oscillate in and out severely, dissipating great kinetic energy of air via viscous boundary layer at perforations. As a result, the energy of sound incipient on top facesheet is largely absorbed by the hybrid metamaterial rather than transmitted or reflected. On account of the tiny influence of incident angle in the frequency range lower than the first sound absorption peak frequency^32^, the oblique incidence case almost maintains the same absorption profile as the normal incident case in the considered frequency range, which has been demonstrated by our numerical simulations (results not shown here for brevity). The following discussion will therefore only normal incidence cases.

**Figure 2:**
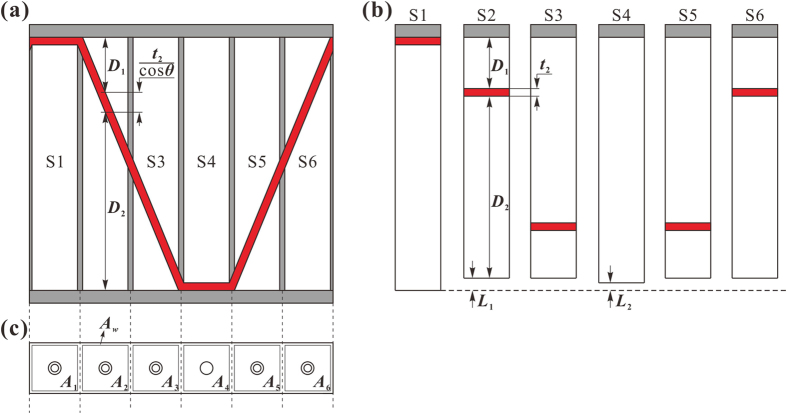
(**a**) Two-dimensional sketch of one unit cell in perforated honeycomb-corrugation hybrid (PHCH). (**b**) Simplified model of six sections in the PHCH by replacing oblique corrugations as horizontal panels. (**c**) Top view of one unit cell in the PHCH. Gray areas refer to honeycomb wall and facesheets, while red areas refer to corrugation.

Based on the two-dimensional (2D) geometrical model of one unit cell shown schematically in Fig. 2, an approximate analytical model is developed. In the geometrical model, while section S4 is a typical single-layer MPP, sections S2, S3, S5 and S6 may be considered as double-layer MPP if the inclined corrugation is treated as horizontal for simplicity. Section S1 is a special case of double-layer MPP with zero-depth of the first cavity. Consequently, the unit cell of PHCH can be visualized as a combination of series of double-layer MPPs, including one typical single-layer MPP and five simplified double-layer MPPs, as shown in Fig. 2(b). It is obvious that S2 is actually the inverse of S3: the thickness of the upper honeycomb cavity in S2 is equal to that of the lower honeycomb cavity in S3. The same applies to the lower honeycomb cavity in S2 and the upper honeycomb cavity in S3. Also, as the pair (S2, S3) is identical to the pair (S6, S5), the calculation results for the former are applicable to the latter. As shown in Fig. 2, there will be cavity losses between the thickness of the PHCH structure and its simplified structure when the horizontal panel replaces the inclined corrugation. The vertical dimension of the corrugation is *t*_2_/cos *θ*, and the vertical dimension of the horizontal panel is *t*_2_, and hence the corresponding cavity loss is *L*_1_ = *t*_2_/cos *θ* − *t*_2_, *L*_2_ = *t*_2_. The equivalent structure shown in Fig. 2(b) has taken into account this cavity loss and will be used next to develop the analytical model.

Sound absorption coefficient at normal incidence of plane acoustic wave can be theoretically obtained by deriving the acoustic impedance of the structure. As shown in Fig. 2, the hybrid structure is actually a series-parallel connection system of the acoustic elements (MPP and Cavity). The acoustic impedance of each series-connection subsection will be first calculated, upon which the total acoustic impedance can be obtained by applying the parallel-connection rule. To acquire the acoustic impedance of each unit cell (i.e., S1–S6) in PHCH, the series-connection rule is first utilized. The acoustic impedance at the input of the *n*th series-connection MPP-Cavity layer^33^,^34^ is given by:


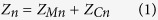


where *n* = 1, 2, …, *N* − 1 with *N* being the total number of the layer, *Z*_*Mn*_ and *Z*_*Cn*_ are the acoustic impedance of the MPP and Cavity of the *n*th layer, that is, the subscripts *M* and *C* represent the initial of the MPP and Cavity, respectively. The acoustic impedance *Z*_*Cn*_ of the *n*th cavity is related to the acoustic impedance *Z*_*n*+1_ of the (*n* + 1)th layer


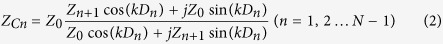


Here *D*_*n*_ is the cavity thickness of the *n*th layer, *Z*_0_ = *ρ*_0_*c*_0_ is the characteristic impedance of air (*ρ*_0_ is the density of air and *c*_0_ the sound speed in air), and *k* = *ω/c*_0_ is the acoustic wavenumber (*ω* is the angular frequency of sound).

For the bottom cavity, its acoustic impedance is


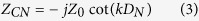


For the acoustic impedance of a small circular tube (perforation), Maa proposed his modified equation based on the solution of Crandall^35^, as^36^,^37^:


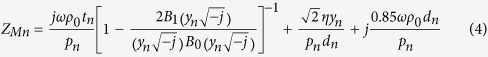


where (*t*_*n*_, *d*_*n*_, *p*_*n*_) are the thickness, perforation diameter and porosity of the nth perforated panel, respectively. 
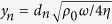
 is 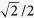 times the ratio of perforation diameter to thickness of viscous boundary layer 
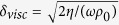
, *η* being the dynamic viscosity coefficient. *B*_*n*_ is the nth order Bessel function of the first kind. The last two terms in Eq. (4) refer to the end corrections in acoustic resistance and reactance, always applied when the tube length *t*_*n*_ is relatively small in comparison with the tube diameter *d*_*n*_.

Employing the above series-connection rule, we can obtain the final acoustic impedance *Z*_1_ at the top surface of each unit cell (i.e., S1–S6), which are re-marked as *Z*_*Sm*_ with *m* = 1, 2, … 6, for the unit cell S1, S2, …, S6, respectively. Since the acoustic impedance of each unit cell *Z*_*Sm*_ (*m* = 1, 2, … 6) is known, the resultant acoustic impedance can be calculated by applying the parallel-connection rule^38^, as:


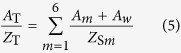


with


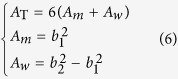


where *A*_T_ and *Z*_T_ are separately the total cross-sectional area of the absorber and the total acoustic impedance of the absorber. *A*_*m*_ (*m* = 1, 2, …, 6) and *A*_*w*_ are the inside area and the outside area of the rectangular core, as shown in Figs 1(a) and 2(c), respectively.

When the honeycomb wall has a non-negligible thickness, a modifying factor of cross-section areas is introduced as:


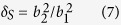


where *b*_1_ and *b*_2_ are the inside and outside length of one rectangular core, as shown in Fig. 1(a). *δ*_*S*_ is actually an indicator referring to the thickness of honeycomb wall. Finally, combining equations (5) and (6) and introducing the modifying factor *δ*_*S*_, we obtain the total acoustic impedance of the PHCH structure as:


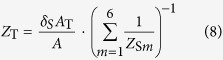


Correspondingly, its sound absorption coefficient is:


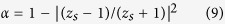


where *z*_*s*_ = *Z*_T_/*Z*_0_ denotes the relative acoustic impedance.

## Results

### Broadband sound absorption at low frequencies

**Figure 3:**
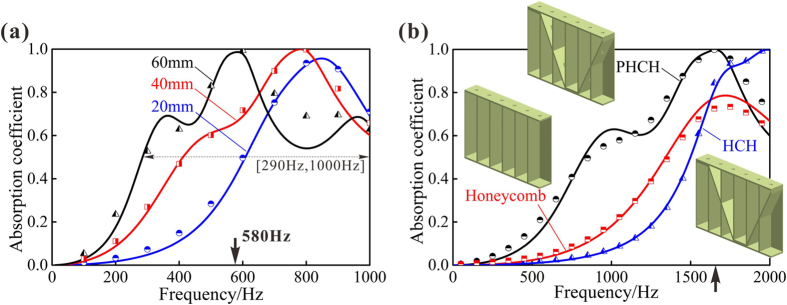
(**a**) Sound absorption coefficient of perforated honeycomb-corrugation hybrid (PHCH) having different H-C hybrid core thicknesses (sample A1: *H* = 20 mm, sample A2: *H* = 40 mm, sample A3: *H* = 60 mm). (**b**) Sound absorption coefficient of PHCH compared with competing structures (sample B). Black, red and blue lines (or markers) represent PHCH, honeycomb and HCH, respectively. All the solid lines correspond to analytical model predictions, while all the markers correspond to finite element (FE) simulation results. Detailed geometric dimensions of all samples are presented in Supplementary Information (SI).

We start by varying the thickness of PHCH to achieve superior sound absorption at low frequencies. Given a specific value of H-C hybrid core thickness *H*, absorbers with different geometrical parameters (*t*_1_, *t*_2_, *d*_1_, *d*_2_) behave distinguishingly. Previous research has also demonstrated higher flow resistances in micro scale flow than in normal scale flow^39^, so the perforation diameters are submillimeter-scale to millimeter-scale. As can be seen from Fig. 3(a), theoretical predictions for samples A1, A2 and A3 agree well with FE simulation results, except for sample A3 at around 800 Hz. These small errors are caused by assuming the inclined corrugation to be horizontal (Fig. 2). For the three samples A1, A2 and A3, the corresponding thickness of viscous boundary layer in the perforation hole is 0.07 mm, 0.08 mm and 0.12 mm, which is smaller than their perforation radius 0.12 mm, 0.14 mm and 0.2 mm, respectively. When the incident sound frequency is low enough, the viscous boundary layer may close up the perforation hole but still cannot change the inherent absorption peak frequency of the structure. As the thickness *H* of the hybrid metamaterial is increased, the sound absorption curve gradually moves from high frequency to low frequency, leading to an absorption peak at 580 Hz (black arrow) when *H* = 60 mm. Moreover, excellent broadband sound absorption can also be achieved at low frequencies, such as a near two-octave 0.5 absorption bandwidth (the bandwidth of whose absorption coefficient is above 0.5) that starts as low as 290 Hz when *H* = 60 mm. Actually, this is a quite good result by such a 60 mm thickness material, given the fact that the corresponding wavelength in air at this low frequency is nearly 1 m. Therefore, this is a real subwavelength acoustic metamaterial as the normalized thickness (i.e., ratio of thickness to wavelength of first peak absorption) is 1/10, 1/11 and 1/20 for the three samples, respectively. Such achievement becomes even more remarkable when considering the fact that the acoustic metamaterial also has outstanding mechanical stiffness and strength. That is, the proposed hybrid structure not only can carry large mechanical load at minimal weight but also can absorb large amount of sound at low frequencies, attractive for applications requiring simultaneous load bearing and sound absorption.

To demonstrate the sound absorption superiority of PHCH and the necessity to perforate the corrugation, three different structures are compared, *i.e*., PHCH, HCH and honeycomb. The PHCH has perforations on both the top facesheet and corrugation, while both the honeycomb corrugation hybrid (HCH) and honeycomb sandwich have perforations only on top facesheet, as shown in Fig. 3(b). With no perforations on corrugation, HCH is divided by the corrugation into two separate parts, of which the lower part does not participate in the acoustical process under the premise of rigidity assumption. Honeycomb contains no corrugated structure, so every section cut apart by the honeycomb wall is identical. Using the analytical model proposed above, we can also evaluate the sound absorption performance of HCH and honeycomb. Figure 3(b) compares the analytical predictions with FE simulation results for the sound absorption of PHCH, HCH and honeycomb. This comparison indicates good agreement between theoretical values and numerical values for all three structures. Regarding sound absorption under 2000 Hz, PHCH shows an appreciable superiority over HCH and honeycomb. Relative to HCH, it is obvious that this advantage at low frequencies is brought in by perforation on corrugation, which introduces one more sound absorption peak related to the second series-wound MPPs. With the best low-frequency absorption, the average absorption coefficient of PHCH is 0.494, which demonstrates a 77.1% enhancement compared to HCH and a 58.3% enhancement to honeycomb. Therefore, this new kind of hybrid acoustic metamaterial shows promising prospects in broadband low-frequency noise control engineering that requires thin thickness and excellent mechanical stiffness/strength and impact energy absorption ability as well.

**Figure 4:**
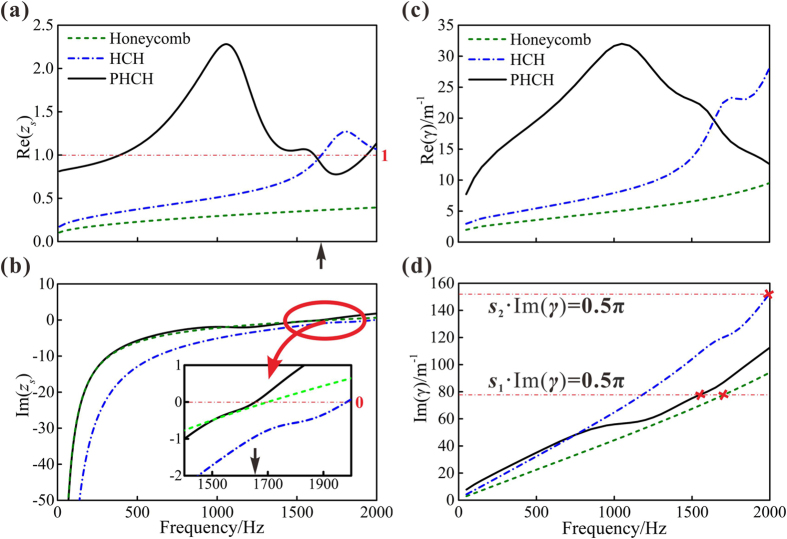
Relative acoustic impedance and propagation constant of PHCH, HCH and honeycomb. HCH and honeycomb have the same dimensions with those of PHCH. The analytically predicted results for: (**a**) Real part of relative impedance and (**b**) Imaginary part of relative impedance. The FE simulation results for: (**c**) Real part of propagation constant and (**d**) Imaginary part of propagation constant. Geometric dimensions of all samples can be found in Supplementary Information (SI).

The sound absorption property of the metamaterial is tightly related to its surface acoustic impedance. According to the theoretical expression for sound absorption coefficient in Eq. (9), perfect sound absorption demands zero reactance (*i.e*., Im(*z*_*s*_) = 0) and resistance equaling to that of the air (*i.e*., Re(*z*_*s*_) = 1) at the same time, *z*_*s*_ = *Z*_T_/*Z*_0_ being the relative acoustic impedance. From the predicted results of relative impedance in Fig. 4, we can see that the reactance goes through zero point at around 1650 Hz while the relative resistance is 0.900, which is very close to one as marked by black arrows on the *x*-axis in Figs 3(b) and 4(a,b). If neither of reactance and resistance satisfies perfect sound absorption requirement, the maximal absorption will not reach one. Such an example is the absorption curve of honeycomb. Comparing honeycomb with HCH in Fig. 4(a), we can see that introducing the corrugation produces a better resistance matching. The relative reactance, which has a positive correlation with the thickness of the backing cavity in MPP, is more important than relative resistance because it has a larger quantity in the low frequency range. Without perforations on the corrugation, the effective thickness of the backing cavity in HCH is clearly reduced compared to PHCH and honeycomb, making the reactance farther from perfect sound absorption demand as shown in Fig. 4(b). These noticeable features of the calculated resistance and reactance indicate the significant roles of the corrugation and the perforations on it, which lead to a better impedance matching of PHCH at low frequencies.

Consider next the propagation constant. As the real part of propagation constant is the attenuation coefficient, Fig. 4(c) illustrates that the sound wave attenuates much more rapidly in PHCH than in either HCH or honeycomb at low frequencies. The imaginary part of propagation constant is also investigated. As is well known, quarter-wavelength resonance will happen in a Helmholtz resonance structure when its thickness equals to one fourth of the sound wavelength. In other word, the first absorption peak shows up when *k* · *s* = π/2, *k* = Im(*γ*) and *s* being the wave number and thickness, respectively. Let *s*_1_ denote the thickness of PHCH and honeycomb. Considering the structural feature of HCH, we use the effective thickness *s*_2_ for calculating, which is the averaged distance from the corrugation to the perforated facesheet. The first absorption peaks marked by red crosses in Fig. 4(d) are in agreement with the results of Fig. 3(b). Obviously, PHCH has a larger wave number than honeycomb so that it can reach quarter-wavelength resonance at a relatively lower frequency. In general, perforations and the corrugation together increase the sound attenuation and decrease the frequency of the first absorption peak, resulting in a better low-frequency absorption performance.

### Energy dissipation modes

**Figure 5:**
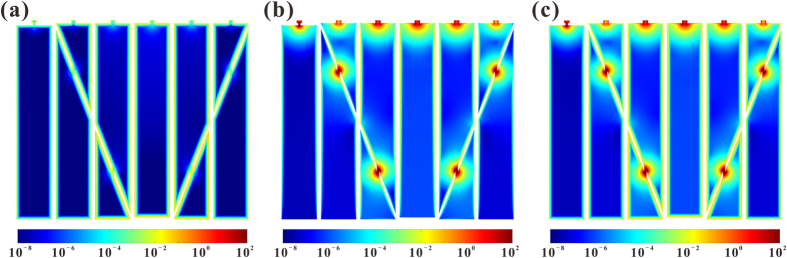
2D contour of energy dissipation at central sectional plane of Sample B (Table S1 of Supplementary Information) at 1650 Hz for (**a**) thermal energy dissipation, (**b**) viscous energy dissipation and (**c**) total viscous-thermal dissipation. The energy dissipation has a unit of W/m^3^. The bottom facesheet thickness *T* = 1 mm.

To further explore the mechanism underlying the sound absorption performance of PHCH, comparisons among different ways of acoustic energy dissipation in one unit cell of PHCH, *i.e*., viscous energy dissipation, thermal energy dissipation and total viscous-thermal dissipation, are performed. The colored area in Fig. 5 refers to the air inside the PHCH structure, which is partitioned into six sections: S1, S2, S3, S4, S5 and S6. Narrow colored areas represent the air in perforations, while the wide colored areas signify the air in honeycomb cavity. It is clear that thermal dissipation, which distributes widely on the inner wall surface, in PHCH is negligible compared to viscous dissipation. Actually, after statistically calculating thermal and viscous energy dissipations in the whole structure, we found that the former one accounted for only 0.04% of total energy dissipation. Therefore, the sound absorption of PHCH is dominated by viscous effect. In Maa’s theory for MPPs, thermal effect is not taken into consideration. It means that the proposed approximation theory based on Maa’s theory only focuses on viscous effect. On account that viscous energy dissipation takes up 99.96% of total energy dissipation, this theory is rational and precise enough for sound absorption characterization. Besides, we found that energy dissipation mainly occurs in narrow areas so that the perforations consume most of the acoustic energy. In fact, one perforation and the honeycomb cavity below it constitute a Helmholtz resonator. When the frequency of sound approaches the resonant frequency, the air in narrow areas oscillates severely. Then the friction between the air and the inner wall of perforation dissipates the kinetic energy of the sound wave remarkably, causing great energy loss in these areas.

### Influence of geometrical parameters

**Figure 6:**
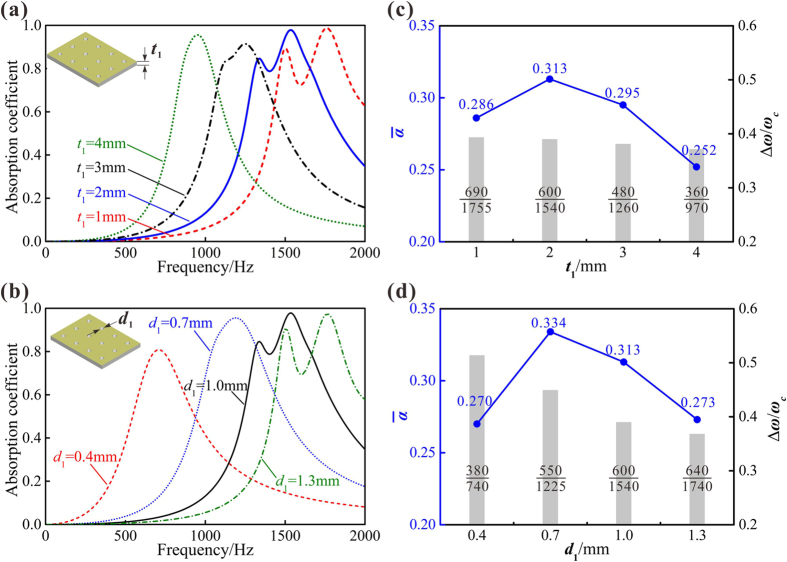
Effects of facesheet geometric parameters on sound absorption of PHCH: (**a**) facesheet thickness *t*_1_ = 1 mm, 2 mm, 3 mm and 4 mm and (**b**) facesheet perforation diameter *d*_1_ = 0.4 mm, 0.7 mm, 1 mm and 1.3 mm. The average absorption coefficient 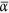 and Δ*ω/ω*_*c*_ of PHCH affected by: (**c**) facesheet thickness *t*_1_ and (**d**) facesheet perforation diameter *d*_1_, here Δ*ω* and *ω*_*c*_ represents the 0.5 absorption bandwidth and the central frequency of the 0.5 absorption band, respectively. Other geometric dimensions (*t*_1_, *d*_1_, *t*_2_, *d*_2_, *b*_1_, *b*_2_, *H, T*) = (2 mm, 1 mm, 1 mm, 1 mm, 3.6 mm, 4 mm, 20 mm, 1 mm) when not being studied as a variation.

To understand the influence of key geometrical parameters on absorption performance of PHCH, varied scales for thicknesses and perforation diameters of the facesheet and the corrugation are investigated. Using our theoretical model, we obtain a relationship between these parameters and absorption performance at low and intermediate frequencies. Absorption curves of PHCH with selected values of facesheet thickness and facesheet perforation diameter are displayed in Fig. 6. The average absorption coefficient and the 0.5 absorption bandwidth are plotted in Fig. 6(c) for different facesheet thicknesses, and plotted in Fig. 6(d) for different facesheet perforation diameters. On the one hand, with the facesheet thickness increasing and the facesheet perforation diameter decreasing, the two absorption peaks come closer to each other, ending in one single visible peak at lower frequencies. Since the total sound absorption is contributed by the six sections (see Fig. 2), its peaks are induced by the absorption peak of each section. At thicker facesheet thickness and smaller perforation diameter, the resonant frequencies of the six sections become lower and less discrete, thus contributing to just one total absorption peak at lower frequencies. On the other hand, for frequencies below 2000 Hz as considered in the present study, the 0.5 absorption bandwidth tends to narrow down when the facesheet thickness increases or the facesheet perforation diameter decreases, except for the *t*_1_ = 1 mm curve in Fig. 6(a) and the *d*_1_ = 1.3 mm curve in Fig. 6(b). However, broadening the bandwidth and enhancing the average absorption calls for relatively thinner facesheet (see Fig. 6(c)), while enhancing the absorption at low frequencies requests relatively thicker facesheet (see Fig. 6(a)). The same principle applies perfectly to the facesheet perforation diameter as low-frequency absorption and broad bandwidth cannot be obtained simultaneously, either. Another method to define bandwidth by introducing coefficient Δ*ω/ω*_*c*_, in which Δ*ω* is the 0.5 absorption bandwidth and *ω*_*c*_ is the central frequency of this bandwidth, can emphasize the importance of low-frequency absorption. Although the 0.5 absorption bandwidth Δ*ω* can be broadened as the facesheet thickness is decreased and the facesheet perforation diameter is increased, the central frequency *ω*_*c*_ also becomes higher. Further, Δ*ω/ω*_*c*_ remains almost constant as the facesheet thickness is varied, while it becomes bigger when the facesheet perforation diameter shrinks. It proves that broadband low-frequency absorption always requires sub-millimeter perforations.

**Figure 7:**
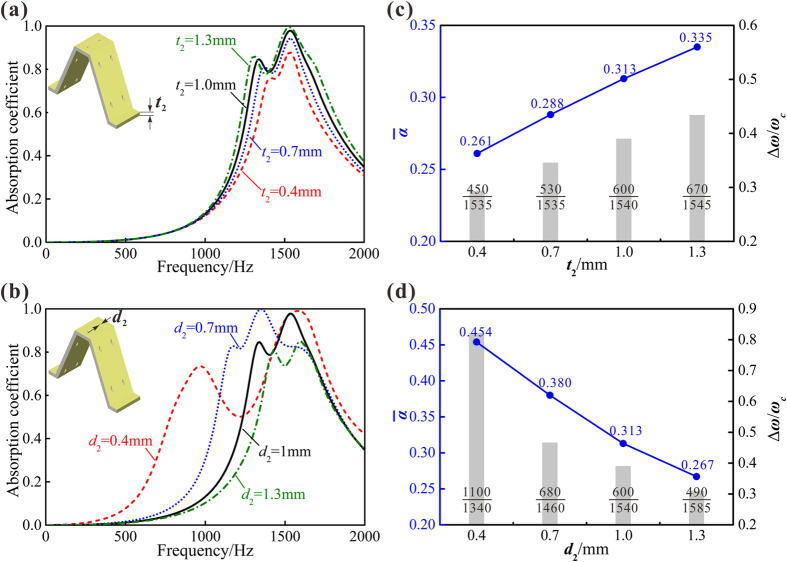
Effects of corrugation geometric parameters on sound absorption of PHCH: (**a**) corrugation thickness *t*_2_ = 0.4 mm, 0.7 mm, 1 mm and 1.3 mm; (**b**) perforation diameter of corrugation *d*_2_ = 0.4 mm, 0.7 mm, 1 mm and 1.3 mm. The average absorption coefficient 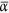 and Δ*ω/ω*_*c*_ of PHCH affected by: (**c**) corrugation thickness *t*_2_ and (**d**) perforation diameter of corrugation *d*_2_, here Δ*ω* and *ω*_*c*_ represents the 0.5 absorption bandwidth and the central frequency of the 0.5 absorption band, respectively. Other geometric dimensions (*t*_1_, *d*_1_, *t*_2_, *d*_2_, *b*_1_, *b*_2_, *H, T*) = (2 mm, 1 mm, 1 mm, 1 mm, 3.6 mm, 4 mm, 20 mm, 1 mm) when not being studied as a variation.

Compared to the facesheet thickness and facesheet perforation diameter, both the corrugation thickness and the perforation diameter of corrugation are proved to have much less influences on the absorption peak, as revealed in Fig. 7. The distance between the first two peaks seems to be apparently enlarged when the perforation diameter of corrugation is reduced and the corrugation thickness increases. However, the variation of peak value is relatively diminutive compared to the facesheet perforation diameter and the facesheet thickness.

To further investigate the effects of corrugation parameters, the average sound absorption coefficient and the 0.5 absorption bandwidth are calculated, as shown in Fig. 7(c,d). In contrast to ignorable alternations of peak values, the average absorption coefficient increases significantly with the corrugation thickening and the perforation diameter diminishing. At the same time, the 0.5 absorption bandwidth broadens and Δ*ω/ω*_*c*_ becomes bigger. In other words, though the corrugation thickness and the perforation diameter of corrugation have less influences on the frequencies of absorption peaks, these parameters can still change the bandwidth and overall absorption performance considerably. In comparison with the facesheet, the average absorption coefficient, the 0.5 absorption bandwidth and the first absorption peak frequency of corrugation have consistent variation with the two investigated parameters. When we increase the corrugation thickness or decrease the perforation diameter of corrugation, all the three reference quantities indicate a better absorption property at low and intermediate frequencies.

For the purpose of gaining excellent sound absorption at low and intermediate frequencies, ordinary MPPs always have a relatively thin perforated panel with submillimeter scale of perforation and a relatively thick backing cavity. Given their complex and paradoxical contribution to low-frequency absorption performance, new types of hybrid structure should be designed to satisfy with diverse demands in specific conditions, especially when non-acoustic performance is also of concern.

## Discussion

Reducing noise effectively and efficiently requires limited thickness and perfect sound absorption performance in broadband frequencies, especially in low frequencies. We have proposed a novel class of hybrid acoustic metamaterial based on lightweight sandwich panel with perforated honeycomb-corrugation core, and demonstrated it has outstanding sound absorption over a broadband low frequency range, as well as excellent mechanical performance. Making use of the electro-acoustical circuit analogy method the traditional theory for MPPs, we developed an approximation theory to compute its sound absorption coefficient. Theoretical and numerical results show a good agreement, demonstrating that this new kind of sound absorber with 60 mm thickness can achieve perfect absorption around 580 Hz, with a broadband absorption bandwidth. The sample with 20 mm thickness has 77.1% advancement over those without perforated corrugation and 58.3% over honeycomb sandwich in low-and-intermediate frequency range in the aspect of average absorption. Discussion about energy dissipation mechanisms validates that viscous dissipation dominates while thermal dissipation can be neglected. Strong energy dissipation in perforations is attributed to the Helmholtz resonance effect. Further analysis shows that the impacts of the perforation diameter and the thickness of facesheet are greater than those of corrugation. Systematic variation of key geometric parameters illustrates that good sound absorption performance in low-and-intermediate frequency range calls for submillimeter scale of perforation diameters on both the facesheet and the corrugation. The theory and results in this work enable designing new broadband sound absorbers that also possess great mechanical performance.

## Methods

### Numerical simulations

**Figure 8:**
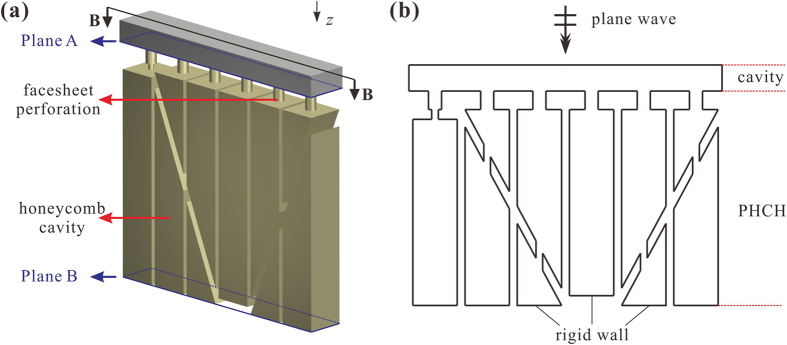
Simulation setup used to analyze the sound absorption coefficient and acoustic impedance of PHCH: (**a**) simulation setup and (**b**) 2D plot from cut plane B-B.

The sound absorption coefficient and acoustic impedance are compared to virtual measurements obtained from FEM simulations using COMSOL Multiphysics. The 3D model shown in Fig. 8(a) is used to simulate the air in PHCH, in order to gain the pressure field and velocity field inside the structure. The whole model can be divided into two parts as depicted in Fig. 8(b): one is the cavity on the top, using the Pressure Acoustics physics model; the other is the PHCH on the bottom, using the Thermoacoustics physics model. Rigid walls (*i.e*., Sound Hard Boundary) are applied to simulate the rigid structure and periodic arrangements in the transverse direction. Critical field variables are the pressure field and the velocity field in the incident interface, which is the blued area Plane A in the figure. The relative acoustic impedance can be given as:


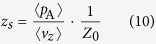


in which 〈*p*〉 and 〈*v*_*z*_〉 denote the average pressure and velocity, respectively, and 〈·〉 represents the area-averaging manipulation over plane A [Fig. 8(a)].

The fluid in and around the absorber is air with mass density *ρ*_0_ = 1.213 kg/m^3^, sound speed *c*_0_ = 343 m/s and dynamic viscosity *η* = 1.814 × 10^−5^ Pa · s.

The propagation constant can be obtained by:


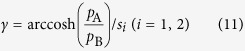


in which *s*_1_ = *t*_1_ + *D* for PHCH and honeycomb and 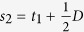 for HCH.

## Additional Information

**How to cite this article:** Tang, Y. *et al*. Hybrid acoustic metamaterial as super absorber for broadband low-frequency sound. *Sci. Rep.* **7**, 43340; doi: 10.1038/srep43340 (2017).

**Publisher's note:** Springer Nature remains neutral with regard to jurisdictional claims in published maps and institutional affiliations.

## Supplementary Material

Supplementary Information
