# Erratum: Hybrid acoustic metamaterial as super absorber for broadband low-frequency sound

**DOI:** 10.1038/srep44972

**Published:** 2017-03-28

**Authors:** Yufan Tang, Shuwei Ren, Han Meng, Fengxian Xin, Lixi Huang, Tianning Chen, Chuanzeng Zhang, Tian Jian Lu

Scientific Reports 7: Article number: 4334010.1038/srep43340; published online: 02 27 2017; updated: 03 28 2017

The original version of this Article contained an error in the spelling of author Tian Jian Lu, which was given as Tian Jain Lu.

This has now been corrected in the HTML and PDF versions of this Article.

