# Supplementary material for: Hybrid acoustic metamaterial as super absorber for broadband low-frequency sound
Source: Sci Rep. 2017 Feb 27;7:43340. doi: 10.1038/srep43340 (PMC5327422; doi:10.1038/srep43340)
Supplement: Supplementary Information [file srep43340-s1.pdf]

Supplementary Information for "Hybrid acoustic metamaterial as super absorber for broadband low-frequency sound"

Yufan Tang[1,2], Shuwei Ren[1,2], Han Meng[1,2], Fengxian Xin[1,2,*], Lixi Huang[3], Tianning Chen[4], Chuanzeng Zhang[5], Tian Jian Lu[1,2,*]

[1]*State Key Laboratory for Strength and Vibration of Mechanical Structures, Xi'an Jiaotong University, Xi'an 710049, P.R. China*

[2]*MOE Key Laboratory for Multifunctional Materials and Structures, Xi'an Jiaotong University, Xi'an 710049, P.R. China*

[3]*Department of Mechanical Engineering, The University of Hong Kong, Pokfulam Road, Hong Kong*

[4]*School of Mechanical Engineering, Xi'an Jiaotong University, Xi'an 710049, P.R. China*

[5]*Department of Civil Engineering, University of Siegen, Siegen 57068, Germany*

Table S1

Geometric dimensions for different PHCH samples investigated in the paper.

Sample number	A1	A2	A3	B
H /mm	20	40	60	20
t_1 /mm	2.8	0.45	0.59	0.29
t_2 /mm	0.12	0.23	0.38	0.22
d_1 /mm	0.59	0.66	0.98	0.54
d_2 /mm	0.24	0.28	0.41	0.24
b_1 /mm	3.6	5.6	7.4	3.6
b_2 /mm	4	6	8	4
T /mm	1	1	1	1

* fengxian.xin@gmail.com (F.X. Xin); tjlu@mail.xjtu.edu.cn (T.J. Lu)

Note: Samples A1, A2 and A3 are studied in Figure 3(a). Sample B is studied in Figure 3(b), Figure 4 and Figure 5.
